# Phytochemical Composition, Antioxidant Activity, and the Effect of the Aqueous Extract of Coffee (*Coffea arabica* L.) Bean Residual Press Cake on the Skin Wound Healing

**DOI:** 10.1155/2016/1923754

**Published:** 2016-11-14

**Authors:** Regina Celis Lopes Affonso, Ana Paula Lorenzen Voytena, Simone Fanan, Heloísa Pitz, Daniela Sousa Coelho, Ana Luiza Horstmann, Aline Pereira, Virgílio Gavicho Uarrota, Maria Clara Hillmann, Lucas Andre Calbusch Varela, Rosa Maria Ribeiro-do-Valle, Marcelo Maraschin

**Affiliations:** Plant Morphogenesis and Biochemistry Laboratory, Federal University of Santa Catarina, 1346 Admar Gonzaga Road, 88048-000 Florianópolis, SC, Brazil

## Abstract

The world coffee consumption has been growing for its appreciated taste and its beneficial effects on health. The residual biomass of coffee, originated in the food industry after oil extraction from coffee beans, called coffee beans residual press cake, has attracted interest as a source of compounds with antioxidant activity. This study investigated the chemical composition of aqueous extracts of coffee beans residual press cake (AE), their antioxidant activity, and the effect of topical application on the skin wound healing, in animal model, of hydrogels containing the AE, chlorogenic acid (CGA), allantoin (positive control), and carbopol (negative control). The treatments' performance was compared by measuring the reduction of the wound area, with superior result (*p* < 0.05) for the green coffee AE (78.20%) with respect to roasted coffee AE (53.71%), allantoin (70.83%), and carbopol (23.56%). CGA hydrogels reduced significantly the wound area size on the inflammatory phase, which may be associated with the well known antioxidant and anti-inflammatory actions of that compound. The topic use of the coffee AE studied improved the skin wound healing and points to an interesting biotechnological application of the coffee bean residual press cake.

## 1. Introduction

The imbalance between the production of reactive oxidizing agents and the ability of a biological system to counteract the reactive intermediates results in oxidative stress. The reactive oxidizing agents are formed during normal physiological processes or under stress conditions that can damage biological system [1].

The coffee (*Coffea arabica* L.) beans press cake is a residual biomass from the coffee beans oil extraction process, claimed to be rich in bioactive compounds of interest for human health and cosmetics. There is considerable emphasis on the recovery of plant biomass originating from the food industry in order to target it to other industries [2], adding value and reducing eventual environmental damage. In this sense, the large amount of* C. arabica* beans produced in Brazil gives rise to certain amounts of biomass not acceptable by the beverage market, but with an important potential for the development of other products such as cosmetics.

Coffee has been claimed as a functional beverage being an important source of antioxidants in human diet, especially due to the high amounts of phenolic compounds and caffeine. The chemical constituents of arabica coffee involve phenolic compounds and their derivatives (such as chlorogenic acids), alkaloids (specially caffeine), diterpenoid alcohols (such as cafestol and kahweol), carbohydrates, lipids, and volatile and heterocyclic compounds [3]. Chlorogenic acids (CGA) are esters of the* trans*-cinnamic acids [4] whose amounts in green coffees are significantly reduced during the roasting process [5]. In the past decades, polyphenolic compounds have been proposed as one of the most effective functional ingredients in foods and beverages, with antiaging properties and able to neutralize the effects of oxidative damage to the skin, for example, [6]. Studies with* C. arabica* extracts have revealed a set of important biological activities, for example, antibacterial [7], antiviral [8], anti-inflammatory [9, 10], suppressive activity of metalloproteinase expression [11], and reduction of oxidative damage to macromolecules [12]. Besides phenolic compounds, coffee is well recognized as a rich source of the alkaloids, especially caffeine. Such secondary metabolites have presented relevant biological activities as, for instance, stimulation of the central nervous system, diuretic, and peripheral vasoconstriction [13]. Caffeine is the major alkaloid present in coffee beans and its content is correlated with the quality of the beverage, also contributing to the brew bitterness [14].

Skin serves as a protective barrier against the environment and also plays a fundamental role in the homeostasis maintenance [15]. The loss of the skin integrity results in injury that can lead to serious commitments to the body if not repaired. The tissue lesions trigger intracellular responses that coordinate the restoration of tissue's integrity and homeostasis. The ability to respond to the injury and tissue repair is a fundamental property for multicellular organisms. The cutaneous tissue repair occurs via tissue regeneration, with the recovery of tissue functionality, or the healing, and the restoration of tissue homeostasis [16].

The wound healing is a complex biological process after an injury. This process can be understood into three classic stages: inflammation, proliferation, and remodeling. The inflammatory stage begins immediately after injury of the tissue and lasts for around 3 days with inflammation. Subsequently, there are cellular proliferation, migration of different cell types, angiogenesis, and extracellular matrix components production, especially collagen. The proliferation stage may last from 2 to 10 days and is characterized by the migration and differentiation of various cell types, being the most abundant fibroblasts. The remodeling stage is the third and last stage of repair. It begins 2 to 3 weeks after the injury and lasts for about a year or more. The majority of endothelial cells, myofibroblasts, and macrophages are destroyed by apoptosis and these changes result in wound contraction and scar formation [16]. Inflammation and oxidative stress are closely related at the wound site considering the influx of neutrophils and macrophages producing reactive oxygen and nitrogen. Reactive oxygen and nitrogen species are involved in the redox regulation of cell functions and increasingly viewed as a major upstream component in the signalling cascade involved in inflammatory responses and the stimulation of adhesion molecule and chemoattractant production [15]. Testing by oxygen radical absorbance capacity assay of the coffee berry outperforms common antioxidants such as green tea extract, pomegranate extract, vitamin C, and vitamin E. Polymerase chain reaction microarray analysis on human cultured fibroblasts treated with multiple doses of 0.001% coffee berry revealed upregulation of gene expression for several collagens and connective tissue growth factor and downregulation for metalloproteinases [17].

It is known that the effect of aging causes a temporal delay in wound healing, but not an actual impairment in terms of the quality of healing [18, 19]. Delayed wound healing in the aged is associated with an altered inflammatory response, such as delayed T-cell infiltration into the wound area with alterations in chemokine production and reduced macrophage phagocytic capacity [20]. Retarded reepithelialization, collagen synthesis, and angiogenesis have also been observed in aged mice as compared with young ones [21].

The topical administration of antioxidant compounds on the skin is gaining prominence among dermatologists because of their anti-inflammatory and anticarcinogenic activity [22]. In this context, it has been claimed that wound repair process could be helped by topically administrating antioxidant molecules that may control the oxidative stress [23]. Phenolic compounds have been showed to be active on skin tissues through hampering collagen destruction and collagenase activation [24]. Indeed, CGA has been shown to accelerate the skin wound healing and burn healing due to its antioxidant, free radical scavenging, anti-inflammatory, radioprotective, antiulcerogenic, and analgesic properties [10, 25–29]. Importantly, the effectiveness of polyphenols in the repair of cutaneous tissue is initially determined by their physicochemical properties and the ability to overcome the epidermal barrier to achieve appropriate receptors [6].

In this sense, the working hypothesis of this study is based on the assumption that a biologically compatible extract, that is, the AE of coffee beans residual press cake, is a rich source of polyphenolic compounds that topically administrated on the damaged cutaneous tissue would ameliorate the wound healing process. Thus, the phytochemical profile and the antioxidant activity of the AE of coffee beans (*C. arabica* L.) residual press cake were determined. In a second approach, the* in vivo* effect of topically administrated hydrogels containing AE and chlorogenic acid on the wound healing process was investigated using a skin excision wound model in Swiss albino mice.

## 2. Material and Methods

### 2.1. Coffee Samples, Chemicals, and Cell Lines

Samples of green (i.e., nonroasted beans) and roasted coffee beans press cake residual were supplied by Cooxupé (Regional Cooperative of Coffee Growers Ltda, at Guaxupé, Minas Gerais State, southeastern Brazil). Green and roasted coffee bean press cake samples were obtained after mechanically pressing the beans for the extraction of the oil fraction. After extraction, the residual biomass was packed in polyethylene bags, with silica sachets, and stored at −20°C for further analysis. Analytical grade methanol, ethanol, hydrochloric acid, sodium chloride, and hydrogen peroxide were purchased from Vetec (Rio de Janeiro, Brazil). The Folin-Ciocalteu reagent, sodium dodecyl sulphate (SDS), dimethyl sulfoxide (DMSO), 2,2-diphenyl-1-picrylhydrazyl (DPPH), (±)-6-hydroxy-2,5,7,8-tetramethylchroman-2-carboxylic acid (Trolox), potassium phosphate buffer (TPK), Tween® 20, Bradford reagent, bovine serum albumin (BSA), methionine, riboflavin, nitrotetrazolium blue chloride (NBT), neutral red (NR), and the analytical reference standards (chlorogenic acid, gallic acid,* p*-hydroxybenzoic acid, ferulic acid, caffeic acid, syringic acid,* p-*coumaric acid, caffeine, theophylline, theobromine, trigonelline, and allantoin) were purchased from Sigma-Aldrich (Missouri, USA). Carbopol 940 NF polymer, disodium ethylenediaminetetraacetic acid (EDTA), and aminomethyl propanol (AMP) were obtained from Pharma Nostra (Campinas, Brazil). Mouse fibroblasts L929 cell line was purchased from Rio de Janeiro Cell Bank (BCRJ), as Dulbecco's modified eagle's medium (DMEM), fetal bovine serum (FBS), penicillin, and streptomycin were obtained from Sigma-Aldrich (Missouri, USA).

### 2.2. Preparation of the Aqueous Extracts of Green and Roasted Coffee Bean Residual Press Cake

AE extracts of the biomasses in study were obtained according to the cold extraction method from the Brazilian Pharmacopoeia [30], with modifications. Samples (4 g, dry weight) were added of 100 mL EtOH solution 70% (v/v) at room temperature, followed by magnetic stirring during 1 h, protected from the light, and kept 18 h under refrigeration (2–8°C). The extracts were recovered by filtration on cellulose support under reduced pressure, followed by concentration in a rotatory evaporator (60°C, 82 rpm). Dried extracts were resuspended in 10 mL distilled-deionized water, centrifuge (10 min, 4000 rpm, 13.7 cm rotor radius), and the supernatant collected for further analysis.

### 2.3. Preparation of the Treatments for Topical Application on the Skin Wound Healing

Carbopol hydrogel was prepared containing 1% (w/v) carbopol 940, 0.1% (w/v) EDTA, 0.5% (w/v) aminomethyl propanol, and water (60 mL). The carbopol hydrogels were used as a vehicle base and the treatments were prepared as follows: (1) carbopol base plus 10% AE (v/v) green coffee, (2) carbopol base plus 10% AE (v/v) roasted coffee, (3) carbopol base plus 3% (v/w) chlorogenic acid, (4) carbopol base plus 1% (v/w) allantoin-positive control, and (5) carbopol base-negative control. The chlorogenic acid concentration in the hydrogel (3%, w/v) was chosen according to the content of this compound in the AE of green coffee press cake previously determined by HPLC analysis.

### 2.4. Determination of Total Phenolic Contents

The total phenolic contents of the AE were determined using the Folin-Ciocalteu reagent, according to Randhir et al. [31]. For that, 1 mL AE was diluted in 9 mL distilled-deionized water. The diluted sample (40 *µ*L) was added to 3.16 mL distilled-deionized water and 200 *µ*L Folin-Ciocalteau reagent, vortexed, stirring for 1 min, after which 600 *μ*L of sodium carbonate solution was added (20%, w/v), vortexed, and incubated for 2 h. The blank solution was prepared as described above, by replacing the test sample by distilled-deionized water. The absorbance was measured at 750 *η*m using a UV spectrophotometer (BEL LGS 53, BEL Engineering, Monza, Italy) and the analysis was carried out in triplicate. The results were expressed as mean (mg equivalent gallic acid/g biomass) ± standard error of the mean (sem), based on a calibration curve of gallic acid (100–700 *µ*g·mL^−1^, *r* ^2^ = 0.983, *y* = 0.001*x*).

### 2.5. RP-HPLC Analysis of Phenolic Acids and Alkaloids

The RP-HPLC analysis was adapted from Rodrigues and Bragagnolo [32] that propose simultaneous determination of caffeine and chlorogenic acids. AE aliquots (60 *µ*L) were injected into a liquid chromatograph (HPLC Thermo Scientific UltiMate 3000 RS Dual System) equipped with a reverse-phase column (Thermo Scientific C18, 250 mm × 4.6 mm, Ø 0.5 *µ*m particle, 35°C) and a diode array detector operating at 240 nm, 260 nm, 280 nm, and 320 nm. The alkaloids and phenolic compounds were quantified at 280 nm and 320 nm, respectively, according to previous records. The mobile phase consisted of HCl acidified Milli-Q water (pH 2.3-Eluent A) and methanol (Eluent B). The chromatographic conditions for the gradient were as shown in Table 1.

A flow rate at 1 mL/min and a run time of 50 min were used. Besides, the AE was solubilized in 10 mL 80% methanol and diluted 9 : 1 (v/v) previously to injection. The quantification of phenolic compounds was based on the integration of the peak areas and a calibration curve of chlorogenic acid (detection range 10–400 *µ*g·mL^−1^, *r* ^2^ = 0.99, *y* = 0.707*x*). Similarly, the amounts of alkaloids were determined based on the integration of the peak areas and calibration curve of caffeine (detection range 100–1000 *µ*g·mL^−1^, *r* ^2^ = 0.99, *y* = 0.842*x*). The analysis was carried out in triplicate. The results were expressed as mean (mg·g^−1^) ± standard deviation (sd).

### 2.6. DPPH Radical Scavenging Activity Assay

The AE was firstly freeze-dried, weighted, and resuspended in 95% methanol (v/v) for a final concentration at 10 mg/mL AE dried mass. Antioxidant activity of the green and roasted coffee AE was determined by measuring their scavenging capacity of the 2,2′-diphenyl-1-picrylhydrazyl (DPPH) radical as previously described and adapted by Molyneux [33]. The (±)-6-hydroxy-2,5,7,8-tetramethylchroman-2-carboxylic acid (Trolox) was used as positive control and methanol as the negative one. The amount of 28 *µ*L DPPH methanolic solution (70 *µ*M) was incubated with 972 *µ*L sample test (from 1 to 30 *µ*g/mL), for 20 min, at 25°C (*n* = 3). DPPH scavenging activity, which manifests itself as a decrease in absorbance, was measured at 517 nm with a UV-Vis spectrophotometer (UV-2000 Instruterm). The DPPH radical scavenging activity (%) was calculated as follows:

(1) Radical  scavenging  activity%=100−Abs  sample∗100Abs  negative  control.

The experiment was carried out in triplicate and data are expressed as mean ± standard deviation (sd).

### 2.7. *In Vitro* Cell Viability

The effect of the AE on the cell viability was determined* in vitro* according to the protocol established by ICCVAM [34], with modifications. The method is based on the assessment of cell viability* in vitro* after exposure to the AE, measuring the lysosome's uptake of neutral red dye. For that, mouse L929 fibroblasts were plated in 96-well plates (1 × 10^4^ cells/well) and incubated during 24 h (37°C, 95% humidity, and 5% CO_2_). After this period, cells were washed with phosphate buffered saline (PBS) and treated with increasing concentrations of coffee AE (3, 10, 30, 100, 300, 1000, 3000, and 10000 *µ*g/mL), during 24 h (37°C, 95% humidity, and 5% CO_2_). The protein denaturing agent SDS, in the same concentrations of the coffee AE, was used as positive control. With the treatment period elapsing, cells were washed with PBS and incubated in the presence of neutral red dye (25 *µ*g/mL DMEM) for 3 h (37°C, 95% humidity, and 5% CO_2_). Subsequently, cells were washed with PBS and neutral red dye destaining solution was added (1% acetic acid : 50% ethanol : 49% distilled water, v/v/v). Absorbance was measured at 540 nm using a microplate reader (*SpectraMax Paradigm Multi-Mode Microplate* Reader, Molecular Devices, Sunnyvale, USA). Cell viability was expressed as percentage of growth based on the control cells and concentration of test samples which showed 50% inhibition of cell grow (IC50), expressing the results in mg AE/mL. The results were calculated according to the equation below:

(2) $$\begin{array}{c} \left[\;\left(\;C_{v}\;\right)=\left[\;\frac{\left(C_{me}-B_{m}\right)}{\left({Ctr}_{m}-B_{m}\right)}\;\right]*100\;\right], \end{array}$$

where *C*_*v*_ is the cell viability (%), *C*_me_ is the mean absorbance of each concentration of the sample, *B*_*m*_ is the mean absorbance of the blank, and Ctr_*m*_ is the mean absorbance of control.

### 2.8. *In Vivo* Skin Wound Healing Assays

Swiss albino male mice* (Mus musculus)*, 40–60 g, 9 months old (assay 1), and 2 months old (assay 2), from the Central Biotery of the Federal University of Santa Catarina (Florianópolis, Santa Catarina State, southern Brazil) were adapted to the laboratory conditions prior to the assay, having free access to food and water. The animals were anesthetized using an intraperitoneal injection of 10% ketamine (75 mg/kg) and 2% xylazine (10 mg/kg). The excisional wound model was realized according to Frank and Kämpfer [35]. Briefly, the dorsal surface of the thoracic region was trichotomized and cleaned up with iodized alcohol. A 10 mm diameter skin wound was made with a scissor aid under aseptic conditions. Hydrogels (0.1 g/animal) were applied topically and daily. Carbopol hydrogel (1%, w/v) was used as negative control, as carbopol hydrogel containing allantoin (1%, w/v) was chosen as positive control, because of the recognized antiseptic action and ability to promote cell proliferation and wound healing of that compound [36]. The wound contraction was evaluated and the wound area measured at the end of the experimental period, that is, day 14. The experimental protocol was approved by the Ethics Committee on the use of animals (Federal University of Santa Catarina, PP0957).

#### 2.8.1. Evaluation of Wound Healing 1: Treatment with Hydrogels Containing Green and Roasted Coffee Bean AE

The aim of this experiment was to determine the effect of the topical treatment with AE of green and roasted coffee beans press cake on the skin wound healing. This investigation adopted an* in vivo* assay with 9-month-old mice, in order to verify the wound healing potential of the AE in an animal group with delayed metabolism due to the advanced age. Initially, 24 male, 40–60 g Swiss albino mice were used, randomly divided into 4 groups (*n* = 6) according to the treatments with hydrogel enriched with (1) AE green coffee, (2) AE roasted coffee, (3) allantoin (positive control), and (4) carbopol gel (negative control). Animals were kept in individual cages over a 14-day-long experimental period. Lesions located in the back of the animals were observed and treated daily after the initial surgical procedure to check the reepithelialization process. The wound healing and the efficacy of a treatment were evaluated by assessing the percentage of wound area reduction along the experimental period. The wound area was measured using a digital caliper at the beginning and at the end of the experimental period, calculating the injury reduction percentage. The wound area was recorded and expressed as the percentage reduction in the original wound area according to the equation:

(3) $$\begin{array}{c} \left[\;\left(\;W_{cp}\;\right)=\frac{\left(W_{d0}-W_{dn}\right)}{W_{d0}}*100\;\right], \end{array}$$

where *W*_cp_ is the wound contraction percentage (%), *W*_*d*0_ the wound area on day “0,” and *W*_*dn*_ the wound area on day “*n*.”

#### 2.8.2. Evaluation of Wound Healing 2: Treatment with Hydrogels Containing Chlorogenic Acid and Green Coffee Bean AE

In a second experimental approach, the effectiveness of the topical administration of hydrogels containing chlorogenic acid or AE of green coffee was determined on the skin wound healing, using Swiss albino mice 2 months old. A total of 96 male, 40–60 g Swiss albino mice were randomly divided into 3 groups (*n* = 32); considering different sampling times (3, 7, and 15 days). Subsequently, each time group was separated into 4 groups (*n* = 8) according to the treatments with hydrogel as follows: (1) AE green coffee, (2) CGA, (3) allantoin (positive control), and (4) carbopol gel (negative control). Animals were kept in individual cages over a 15-day-long experimental period. Lesions were observed and treated daily after the initial surgical procedure to check the reepithelialization process. The skin wound was photographed using a digital camera placed in a standard distance from the lesion, immediately after surgery, and at 3rd, 7th, and 15th days. The wound healing process was evaluated by assessing the size of wound reduction in each sampling time. The wound area was measured through scanned photographic images using the software* ImageJ®*. The results were recorded and expressed as measurement of wound area. Data are presented as means of wound area (cm^2^) ± mean standard error (sem).

### 2.9. Determination of Oxidative Stress Markers

Skin wound samples of the second experimental approach were collected for biochemical analyses following the protocols of Bagdas et al. [37] and Pereira [38]. Skin wound samples were collected and the animals sacrificed immediately. Samples were washed in 0.9% (w/v) NaCl and immediately frozen at −80°C until use. Tissue samples were cut into pieces and homogenized (ULTRA-TURRAX homogenizer) in a 2 mL potassium phosphate buffer solution (20 mM, pH 7.4) with TWEEN®20 (1%) and NaCl (150 mM). The samples were vortexed for 10 s and centrifuged (4000 rpm, 15 min, 4°C) and the supernatants recovered for further analysis. Protein contents were determined according to the Bradford method [39]. The catalase (CAT) activity was measured spectrophotometrically (*λ* = 240 nm), following the method described by Pereira [38]. For the test, 25 *µ*L of supernatant was added to 240 *µ*L of potassium phosphate buffer (50 mM, pH 7.0), supplemented with hydrogen peroxide solution (10 mM). The decomposition rate of hydrogen peroxide was measured sequentially in intervals of 20 s, until 10 min. The results were expressed as mmol·min^−1^/mg protein. The superoxide dismutase (SOD) activity was assessed based on the method of Giannopolitis and Ries [40]. The determination considers the enzyme's ability to inhibit the photoreduction of nitrotetrazolium blue chloride (NBT). The activity was determined adding 25 *μ*L sample to 240 *μ*L work solution in 96-well microplates. The work solution was prepared with 13 mM methionine, 75 *μ*M NBT, 100 nM EDTA, and 2 mM riboflavin in potassium phosphate buffer (50 mM, pH 7.8). The reaction was started by lighting the microplate in a chamber composed of fluorescent tubes (15 W), at room temperature. After 5 min incubation, the end of catalysis was forced by the interruption of light. The content of the blue compound (formazan) formed by the photoreduction of NBT was measured spectrophotometrically at 560 nm. The same reaction was prepared and protected from the light. One unit of SOD is defined as the enzyme activity required for 50% inhibition of NBT photoreduction. To calculate the specific activity of the enzyme, it was considered as the percentage of enzymatic inhibition obtained, the sample volume, and the sample protein concentration (*μ*g protein). The results were expressed as U/*μ*g protein. The enzymatic experiments were realized in triplicate and the data expressed as means ± standard deviation (sd).

### 2.10. Statistical Analysis

Data were collected and summarized following statistical analysis using one-way ANOVA and Tukey's test. The results were considered statistically significant when *p* < 0.05. The values were expressed as mean ± sd or mean ± sem, as indicated in tables subtitles.

## 3. Results and Discussions

In this study, AE of residual biomasses from the green and roasted coffee oil industry were chemically characterized and further investigated to determine the effectiveness of hydrogels containing AE and chlorogenic acid on the cutaneous tissue repair process.

### 3.1. Total Phenolic Contents

Table 2 provides the total phenolic contents of AE of green and roasted coffee press cake showing appreciable and statistically different (*p* < 0.05) amounts of that secondary metabolite in the samples. The highest concentration of phenolic compounds was detected in the green coffee AE. Thus, one could speculate that the extraction of the apolar fraction of the green coffee beans press cake (i.e., oil fraction) allows obtaining a high yield of phenolic compounds in the AE of the residual biomass. It is well known that the phenolic content in coffee beans may vary largely according to the species, variety, degree of maturation, postharvest processing, and roasting. After the harvest and along the coffee bean processing, some phenolic compounds can be isomerized, hydrolyzed, or degraded into low molecular weight compounds. The high temperatures of coffee roasting process degrade part of the phenolic compounds [41]. Indeed, lower amounts (*p* < 0.05) of phenolic compounds were found in the AE of roasted coffee bean sample comparatively to the nonroasted one, proving the negative effect of the roasting process on the contents of those bioactive compounds.

### 3.2. Quantitation of Phenolic Acids and Alkaloids

The use of the liquid chromatography with a gradient elution system containing acidified water and methanol as mobile phase is often in the analysis of phenolic compounds [42]. However, the method adapted in this assay, for simultaneous determination of phenolics and alkaloids, allowed identifying analytes for both groups of compounds. The results revealed chlorogenic acid (11.11 mg·g^−1^) and caffeine (4.5 mg·g^−1^) are the majors compounds in the AE of green coffee samples (Table 3). This finding is consistent with previous results for coffee beans as mentioned by several authors [41, 43–45]. Again, considering that phenolic acids and their derivatives can be degraded under high temperatures, one could speculate that the lowest concentration of these acids in the roasted coffee press cake samples results from the thermal treatment of that biomass during the roasting process. The alkaloid contents revealed important amounts of caffeine and trigonelline in both samples, but low concentration of theophylline. The roasted coffee sample showed superior caffeine amounts comparatively to the other studied sample. Theobromine was not detected. Besides, the studied samples seemed to be quite discrepant in their alkaloid contents, suggesting different potentials regarding their biological effects. The large amount of caffeine in the coffee extracts is responsible for several biological activities [46].

### 3.3. Antioxidant Potential: DPPH Assay

Reactive oxygen species (ROS), reactive nitrogen species (RNS), and reactive sulfur species (RSS) might react with lipid, protein, and DNA to cause inflammation, cancer, and ischemia, for instance. DPPH is a stable free radical by virtue of the delocalization of the spare electron over the molecule as a whole, so that the molecules do not dimerise, as would be the case of most of the free radicals. DPPH radical, which shows absorption at 517 nm, has been used as a convenient tool for the radical scavenge assay, which is independent of any enzyme activity [33]. The DPPH radical scavenging activity (%) of the AE in study, calculated after 20 min of incubation, revealed a maximum activity achieved with 30 *μ*g AE/mL, similar to the positive control (Trolox) activity (Table 4). The quantitation of phenolic compounds and alkaloids in samples revealed the presence of secondary metabolites related to the antioxidant capacity, for example, caffeine and chlorogenic acids. It is long known that the roasting process degrades phenolic compounds; however the alkaloids are more resistant and stable, and caffeine has been shown to present expressive antioxidant activity [47]. It is important to note that the method herein used for measuring total phenolic compounds, that is, Folin-Ciocalteau, is based on a redox reaction as it can be considered an evaluation of the antioxidant activity [48]. The oxidative balance in physiological systems is regulated by endogenous and exogenous mechanisms in which the excess of free radicals is related to many diseases [49, 50]. The control of the excess of oxidative molecules includes the intake or topical application of exogenous antioxidants or even molecules that can stimulate the endogenous antioxidant [12]. Thus, the antioxidant activity of coffee bean extracts is related to the presence of several natural constituents and compounds formed during processing, for example, caffeine [51], chlorogenic and hydroxycinnamic acids [52–55], and Maillard reaction products such as melanoidins [54, 55].

### 3.4. Cell Viability Profile

**Figure 1:**
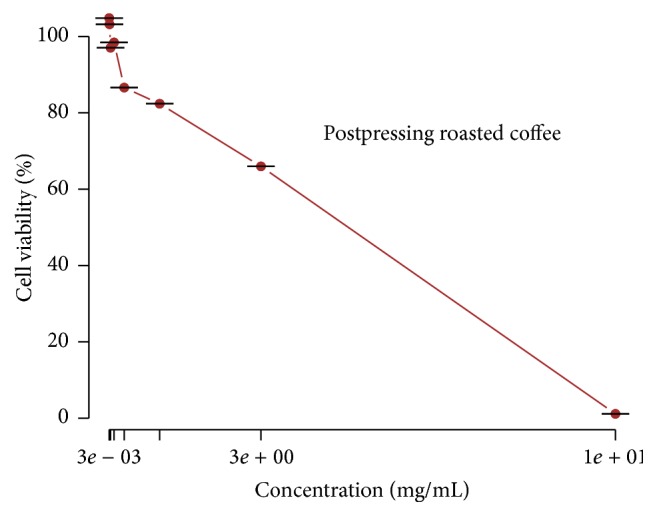
*In vitro* cell viability after 24 h exposure to the AE of roasted coffee press cake. Important reductions of cell viability were detected at AE concentration of 3 mg/mL or higher, as determined through the NRU assay. Results are expressed as mean ± standard deviation (sd).

The cell viability decrease was only detected in cells treated with the roasted coffee AE at 3 mg·mL^−1^, with an IC50 value of 4.88 mg·mL^−1^ and a LD50 = 2482.00 mg·kg^−1^ as showed in Figure 1. Although cell viability decreased totally at 10 mg·mL^−1^, the LD50 value characterized the AE as nontoxic, because the cytotoxicity is considered for LD50 values lower than 2000 mg·kg^−1^. Besides, green coffee AE led to no reduction in cell viability in concentrations equal to or lower than 10 mg/mL. Traditionally,* in vitro* determination of cell viability profile and toxic effect of compounds is the first biological assay to perform aiming at a further application in human health, for instance. If the toxicity of the molecule is not proved, it can proceed to the next step,* in vivo* assay. Experimentally, this has been performed by counting viable cells after staining them with a vital dye. The neutral red assay system is a means of measuring living cells through the uptake of the vital neutral red dye [56] as adopted in this study. As noted by Triglia et al. (1991), the neutral red dye passes through the intact cell membrane and becomes concentrated in lysosomes of viable cells. Test agents that damage the cell surface and lysosomal membrane inhibit the incorporation of the red dye and the amount taken up by the cells is, therefore, proportional to the number of viable ones. The neutral red assay was developed by Borenfreund and Puerner [57] and has been utilized extensively to study the toxic effect of a number of compounds on different cell types grown in monolayer cultures, as L929 cells used in this study. Though cell viability reduction showed the roasted coffee extract, all the extracts concentrations tested in this assay showed being noncytotoxic.

### 3.5. Effect of AE of Green and Roasted Coffee Beans Press Cake on the Wound Healing

**Figure 2:**
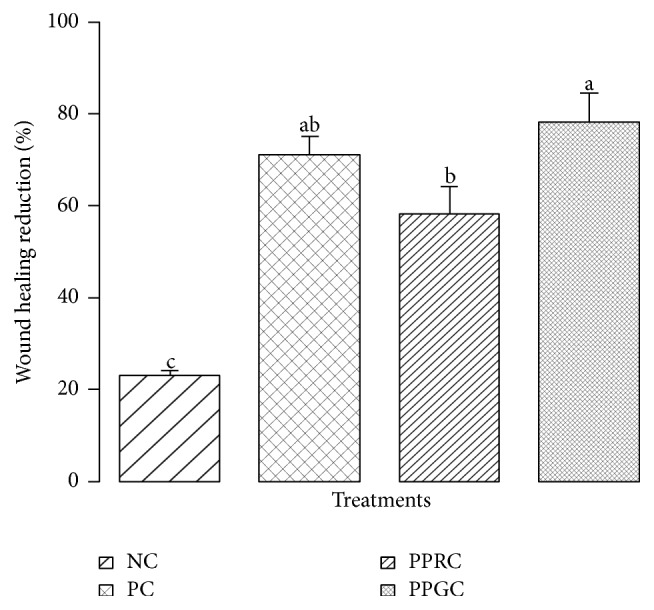
Reduction of the wound area (%) after 14 days of treatment with hydrogels applied daily in mice 9 months old. NC (negative control = 23.56% c), PC (positive control = 70.83% ab), PPGC (postpressing green coffee = 78.20% a), and PPRC (postpressing roasted coffee = 53.71% b). Data are presented as mean ± sem. Distinct letters denote significant differences at *p* < 0.05 (Tukey's test).

The hydrogel containing AE of green coffee showed the best result on the wound reduction (78.20%), being similar to the positive control (70.83%), as can be noted in Figure 2. In its turn, the roasted coffee hydrogel appeared to be less effective in ameliorating the wound healing (53.71%) comparatively to the green coffee AE. A negligible reduction of the wound area was detected for the negative control (23.56%), statistically differing from the other treatments. Thus, it can be assumed that hydrogels enriched with AE of coffee beans press cake improved the wound healing process, revealing the positive effects on the cutaneous tissue regeneration. Indeed, one could argue that the superior performance of the hydrogel containing green coffee extract in the wound healing results from its highest concentration of phenolic compounds according to the phytochemical analysis. Previous* in vivo* assays in mice on the effect of coffee extracts in the skin wound healing process were not found in literature so far. In the present study, it is worth mentioning that by using 9-month-old adult mice, typically differing from other experimental approaches where younger animals have been used [58], the elapsing time of 14 days was not enough to achieve full wound healing, although all treatments significantly decreased the wound area comparatively to negative control. In fact, there are substantial differences in the wound healing response between young and adult subjects, generally lasting longer in adults. The adverse effects of aging on wound repair are well known and were offset by treatments that accelerated the wound closure [59, 60]. Previous study by Bagdas et al. [25] reported that systematical CGA application in skin flap surgeries can accelerate healing and flap survival in animal of wound healing delay by diabetic condition. It has been also suggested that topically CGA ointment has a potent wound healing effect on nondiabetics rats [26]. Thus, our results are consistent with previous reports in literature. In this sense, the findings are relevant since it can be assumed that even for adult individuals the treatment of skin lesions with coffee bean press cake extracts is truly efficient to increase the physiological response, ameliorating the cutaneous regeneration process significantly.

### 3.6. Effect of Chlorogenic Acid on the Wound Healing

The chlorogenic acid hydrogel daily applied on the skin wound appeared to reduce significantly the wound area size in the inflammatory phase (e.g., day 3) which can be associated with its antioxidant and anti-inflammatory activities. Indeed, hydrogel containing CGA resulted (0.57 cm^2^  ± 0.06 sem) in a similar effect regarding the positive control (0.55 cm^2^  ± 0.06 sem) as can be noted in Table 5. Contrarily, the postpressing green coffee AE assayed under the same conditions did not show the same performance (0.75 cm^2^  ± 0.06 sem) at the third day, especially taking into consideration that the wound area at the beginning of the experiment was 0.72 cm^2^  ± 0.02 sem. All the treatments achieved similar performance after 7 days, and 15 days was enough to achieve full wound healing. It can be assumed that the physiological responses in young mice are decisive for full wound healing considering the elapsing time of 15 days. The significant differences at the third day can be attributed to the influence of the treatments on the inflammatory response that begins immediately after injury of the tissue and lasts around 3 days. The inflammation helps wound heal with activation of immune system cellular components, the blood coagulation cascade, cytokines, and the oxidative stress [16]. Studies of Moreira et al. [9] showed that AE of green coffee has anti-inflammatory effect due to the presence of anti-inflammatory and antioxidant compounds. The use of chlorogenic acid improving skin wound healing may be derived from its influence on the inflammatory mediators involved in this response and its antioxidant capacity.

### 3.7. Oxidative Stress Markers

The antioxidant defense system has been developed by the organism as a protective mechanism against ROS formation. Among the most reported endogenous antioxidant systems are the activity of the enzymes SOD and CAT. Wound healing needs a fine balance between the antioxidants activities because ROS are harmful to cells and tissues in cutaneous injury [60]. It is interesting to note the significant increasing of CAT activity in the healed tissues when compared to the unhealed ones (Table 6). Besides, the CAT activity was increased on the tissue treated in all treatment groups along the wound healing process. Probably, the increase can be attributed to the higher ROS levels on the wound bed resulting from the inflammatory response, cellular signaling pathways to avoid the infection, recruiting different kinds of cells, and promoting cells divisions. The increase in the CAT enzymatic activity upon the green coffee AE and hydrogel containing CGA treatments appeared to be increased on day 3 (inflammation stage), following a reduction at the 7th day during the cell proliferation stage, with a strong augment again until day 15. Interestingly, the positive control group also showed a similar profile of CAT activity, except at the end of the experimental period, on day 15. Importantly, regardless of the treatment the results revealed a superior CAT activity along the wound healing process in comparison to the basal level (day 0), suggesting that CAT activity is positively modulated in damaged cutaneous tissues. Finally, meaningful differences (*p* < 0.05) were detected for each sampling time in all treatments, suggesting that a typical standard of CAT activity seems not to occur.

The SOD assay showed higher activity in the early stage of healing (day 3) for the green coffee AE- and CGA-treated groups, followed by a gradual reduction. Furthermore, contrary to CAT activity, SOD decreased bellow the baseline in all experimental groups at the end of the experiment, suggesting that such an enzymatic response to the oxidative stress seems not to be relevant to the cutaneous cells upon regeneration.

## 4. Conclusions

The chemical analysis revealed the green coffee is a raw material richer in chlorogenic acid than the roasted one. The present study shows that the daily topical application of chlorogenic acid and coffee beans press cake AE enhanced the skin wound healing in mice model, especially the later one that reduced more quickly the wound area. Taking into consideration that the experiments were performed with plant extracts, that is, chemically complex matrices, it is assumed that the effects herein described eventually result from the synergistic action of their components. We suggest that the highest amount of CGA and caffeine in the extracts of coffee press cake presents beneficial effects on wound repair by its antioxidant potential, enhancing the physiological responses to achieve the wound closure. The formation of reactive oxygen radical species (ROS) can play an important role in delayed wound healing. Antioxidants can help controlling wound oxidative stress related to ROS generation and thus accelerate wound repair.

Finally, to the best of our knowledge, this is the first study investigating the effect of AE of coffee beans press cake on the skin wound healing, showing beneficial effects on wound repair. Antioxidant and radical scavenger activities of coffee beans press cake AE and CGA can improve wound healing to control overexposure of oxidative stress in the wound bed. The coffee beans press cake has shown a lower commercial value; however it might gain value as thought of as a valuable biomass source of bioactive compounds interesting for the human health usage. Indeed, the residual coffee biomasses studied are able to improve the regeneration of damage skin tissue, allowing new product development in the cosmetical and pharmaceutical industries.

## Figures and Tables

**Table 1:** 

Time (min)	Eluent A (%, v/v)	Eluent B (%, v/v)
0–5	85	15
5–45	0	100
45–50	85	15

**Table 2:** Contents of total phenolic compounds (mg gallic acid equivalents·g^−1^ biomass, dry weight) of the AE of green and roasted coffee beans press cake.

Samples	Total phenolic compounds (mg gallic acid equivalent·g^−1^ biomass, dry weight)
Green coffee	35.39 ± 3.69 a
Roasted coffee	24.13 ± 1.45 b

Mean of extractions in triplicate ± sem. Distinct letters denote significative difference. Tukey's test, *p* < 0.05.

**Table 3:** Concentrations (mg·g^−1^ biomass, dry weight) of phenolic acids and alkaloids of the green and roasted AE of coffee beans determined by RP-HPLC analysis.

^*∗*^Phenolic acid and alkaloids (mg·g^−1^)	Samples	
	Green coffee	Roasted coffee
280 nm		
Chlorogenic acid	11.11 ± 0.28 a	1.95 ± 0.31 b
Syringic acid	0.96 ± 0.04 d	0.84 ± 0.08 c
Ferulic acid	1.40 ± 0.29 d	0.92 ± 0.16 c
Protocatechuic acid	1.20 ± 0.003 d	0.21 ± 0.02 d
Hydroxybenzoic acid	0.08 ± 4.1 e	0.05 ± 1.05 d
Caffeine	4.5 ± 0.06 b	5.60 ± 0.08 a

320 nm		
Caffeic acid	0.01 ± 0.002 e	n.a.
Theophylline	0.01 ± 0.003 e	n.a.
Trigonelline	1.55 ± 0.51 c	1.02 ± 0.14 c

^*∗*^Mean of injections in triplicate ± sd. Distinct letters in the column are significantly different. Tukey's test, *p* < 0.05. n.a. = not available.

**Table 4:** Maximum activity of DPPH radical scavenging of AE of green and roasted coffee beans press cake and Trolox (positive control).

Sample (30 *μ*g·*μ*L^−1^)	Maximum activity (%)^*∗*^
AE green coffee bean press cake	96.21 ± 1.26
AE roasted coffee beans press cake	95.35 ± 2.20
Trolox	96.36 ± 0.33

^*∗*^Values are shown as mean ± standard deviation.

**Table 5:** Wound area size (cm^2^) in the elapsing time of 15 days, with daily application of green coffee press cake and chlorogenic acid hydrogels.

Groups (*n* = 8)	Day 0	Day 3	Day 7	Day 15
GC	0.70 ± 0.02 a	0.75 ± 0.06 ab	0.42 ± 0.04 a	0.002 ± 0.02 a
CGA	0.70 ± 0.02 a	0.57 ± 0.02 a	0.41 ± 0.02 a	0.002 ± 0.02 a
PC	0.72 ± 0.02 a	0.55 ± 0.06 a	0.31 ± 0.04 a	0.00 ± 0.01 a
NC	0.74 ± 0.02 a	0.85 ± 0.06 b	0.42 ± 0.04 a	0.002 ± 0.02 a

Data are presented as means ± sem. Distinct letters in the column denote significant differences (Tukey test, *p* < 0.05). GC = green coffee, CGA = chlorogenic acid, PC = positive control, and CN = negative control.

**Table 6:** Activity of catalase (CAT) and superoxide dismutase (SOD) in the epithelial tissue of Swiss albino mice according to the treatment with AE of green coffee bean press cake and chlorogenic acid related to controls groups.

Day/treatment	GC	CGA	PC	NC
CAT (mmol·min^−1^/mg protein)				
Day 0^*∗*^	164.1 ± 12.6	164.1 ± 12.6	164.1 ± 12.6	164.1 ± 12.6
Day 3	597.5 ± 12.4 b	360.6 ± 12.6 c	909.35 ± 5.8 a	174.7 ± 2.1 d
Day 7	260.7 ± 11.9 d	399.5 ± 19.4 c	846.6 ± 20.1 a	683.7 ± 4.1 b
Day 15	1005.0 ± 16.5 a	506.6 ± 10.7 b	408.7 ± 1.8 c	300.0 ± 12.8 d

SOD (U/mg protein)				
Day 0^*∗*^	0.45 ± 0.02	0.45 ± 0.02	0.45 ± 0.02	0.45 ± 0.02
Day 3	1.05 ± 0.08 b	1.07 ± 0.04 b	0.25 ± 0.01 c	2.33 ± 0.02 a
Day 7	0.52 ± 0.02 a	0.29 ± 0.03 b	0.26 ± 0.02 b	0.20 ± 0.03 c
Day 15	0.12 ± 0.01 b	0.17 ± 0.03 b	0.21 ± 0.02 a	0.25 ± 0.05 a

Data are presented as means ± sd. Different letters in the line denote significant differences (Tukey test, *p* < 0.05). ^*∗*^Day 0 = enzymatic activity on the undamaged tissue. GC = green coffee, CGA = chlorogenic acid, PC = positive control, and CN = negative control.
